# Editorial: Liquid biopsy as a tool for precision oncology: New challenges to assess clinical response, Volume II

**DOI:** 10.3389/fphar.2023.1192549

**Published:** 2023-04-12

**Authors:** Paola Ulivi

**Affiliations:** Biosciences Laboratory, IRCCS Istituto Romagnolo per lo Studio dei Tumori (IRST) “Dino Amadori”, Meldola(FC), Italy

**Keywords:** liquid biopsy, cancer, CTC (circulating tumor cells), cfDNA (cell-free DNA), extracellular vesicles

The availability of tumor tissue represents a starting point for tumor molecular characterization. For some tumor types, including lung cancer, diagnosis is performed on small cytological or biopsy specimens, and there may be few tumor specimens available for nucleic acid extraction and molecular analyses. Moreover, tumor tissue is often heterogeneous, leading to a heterogeneous presence of molecular alterations. In this context, a molecular alteration could be present at one specific site and not at the other. Finally, it is observed that tumors are dynamic; they can evolve during treatment and develop resistance clones, which block treatment and lead to disease progression. This cannot be monitored unless the patient undergoes a second biopsy, subjecting the patient to an invasive procedure. In these contexts, liquid biopsy, as a non-invasive approach, represents a valid alternative for tumor molecular characterization, repeatable several times during patient treatment and representative of overall tumor heterogeneity.

The most common type of liquid biopsy is represented by free circulating DNA (fcDNA) released by tumor cells and which can be quantified and characterized from a molecular point of view as an alternative to tumor tissue. However, other types of liquid biopsy specimens could be used, such as extracellular vesicles (EVs), platelets, and circulating tumor cells (CTCs).

In this Research Topic, four papers reported numerous results in this field. Two papers evaluated the role of fcDNA in the lung and colorectal cancers. In Heeke S et al., the authors described an approach that evaluated EGFR plasma mutations using an automatic cartridge-based PCR system, compared to an FDA-approved assay. The proposed method represented a fast approach for mutation analysis with an obvious advantage for possible use in clinical practice. However, despite 100% specificity, sensitivity was lower than that of the FDA test, with some L858R and exon 19 deletions missed. The authors concluded that the methodology needed improvement to reach higher sensitivity levels before its potential application in clinical practice. In Ulivi P et al., the authors described the clinical utility of fcDNA in colorectal cancer patients. Although there have, so far, been no clinical indications for using liquid biopsy in colorectal cancer patients and given that tumor tissue is usually available from this type of cancer, liquid biopsy could provide complementary information, allowing the tumor clonal evolution to be monitored during treatment. The authors described four clinical cases where liquid biopsy provided valuable information on the unresponsiveness to treatment to orient clinicians for subsequent patient management. They demonstrated that NGS approaches on liquid biopsy are feasible and permit concomitantly the analysis of different gene mutations, providing identification of new alterations arising during treatment.

Although fcDNA analysis allows gene mutations and other types of DNA alterations to be studied, it does not allow for cancer phenotypes, including gene expression of drug targets and protein biomarkers.

Instead, CTCs are intact cells originating from tumors with higher potentialities in terms of molecular characterization. The enumeration of CTCs is a prognostic biomarker, especially for specific tumors such as breast cancer. Moreover, DNA and RNA could be extracted from CTCs, allowing for both mutation and gene expression analyses. Protein biomarkers could also be analyzed on the CTC surface. From the perspective of Kaldijian EP et al., the authors described the potentiality of the RareCyte platform. Not all cancer patients have detectable CTCs, especially if they have an early-stage tumor. So, it is crucial to use highly sensitive platforms. Considering that plasma and CTCs can both be isolated from the same blood sample, through an approach of cfDNA sequencing, followed by CTC sequencing in cases with cfDNA, non-informative samples could be hypothesized to increase the sensitivity.

Another source of liquid biopsy with high potentiality is EVs. EVs are a heterogeneous population of membrane-bound particles released from tumor cells in peripheral blood. These particles are involved in determining the microenvironment composition and pre-metastatic niche, and they contain tumor material that contributes to metastatization and resistance to therapy. Analysis and characterization of the nucleic acids found in EVs could have important clinical implications for the molecular characterization of tumors, especially for RNA fusion detection. However, there are some technical challenges to be solved for potential use in a clinical setting. There is a need to standardize their extraction and characterization. All these aspects are discussed in the review by Goričar et al.

